# Comparative proteomic and clinicopathological analysis of breast adenoid cystic carcinoma and basal-like triple-negative breast cancer

**DOI:** 10.3389/fmed.2022.943887

**Published:** 2022-07-28

**Authors:** Qian Yao, Wei Hou, Junbing Chen, Yanhua Bai, Mengping Long, Xiaozheng Huang, Chen Zhao, Lixin Zhou, Dongfeng Niu

**Affiliations:** ^1^Department of Pathology, Key Laboratory of Carcinogenesis and Translational Research (Ministry of Education), Peking University Cancer Hospital and Institute, Beijing, China; ^2^Gastrointestinal Cancer Center, Key Laboratory of Carcinogenesis and Translational Research (Ministry of Education), Peking University Cancer Hospital and Institute, Beijing, China

**Keywords:** bioinformatics, proteomics, breast cancer biology, adenoid cystic carcinoma, triple-negative breast cancer

## Abstract

**Background:**

Adenoid cystic carcinoma (ACC) is a rare type of triple-negative breast cancer that has an indolent clinical behavior. Given the substantial overlapping morphological, immunohistochemical, and molecular features with other basal-like triple-negative breast cancer (BL-TNBC), accurate diagnosis of ACC is crucial for effective clinical treatment. The integrative analysis of the proteome and clinicopathological characteristics may help to distinguish these two neoplasms and provide a deep understanding on biological behaviors and potential target therapy of ACC.

**Methods:**

We applied mass spectrometry-based quantitative proteomics to analyze the protein expression in paired tumor and adjacent normal breast tissue of five ACC and five BL-TNBC. Bioinformatic analyses and the clinicopathological characteristics, including histological features, immunohistochemistry, and FISH results, were also collected to get comprehensive information.

**Results:**

A total of 307 differentially expressed proteins (DEPs) were identified between ACC and BL-TNBC. Clustering analysis of DEPs clearly separated ACC from BL-TNBC. GSEA found downregulation of the immune response of ACC compared with BL-TNBC, which is consistent with the negative PD-L1 expression of ACC. Vesicle-mediated transport was also inhibited, while ECM organization was enriched in ACC. The top upregulated proteins in DEPs were ITGB4, VCAN, and DPT. Moreover, in comparison with normal breast tissue, ACC showed elevated ribosome biogenesis and RNA splicing activity.

**Conclusion:**

This study provides evidence that ACC presents a substantially different proteomic profile compared with BL-TNBC and promotes our understanding on the molecular mechanisms and biological processes of ACC, which might be useful for differential diagnosis and anticancer strategy.

## Introduction

Adenoid cystic carcinoma (ACC) is a malignant tumor that mostly occurs in the salivary glands, which can also affect other anatomical sites, such as the orbit, tracheobronchial tree, prostate, esophagus, and breast (1–5). Primary ACC of the breast is a rare form of invasive carcinoma comprising <0.1% of breast carcinomas (6). Breast ACC demonstrates a basal-like triple-negative (BL-TNBC) phenotype, which negatively expresses estrogen receptor (ER), progesterone receptor (PR), and human epidermal growth factor receptor type 2 (HER2) and positively expresses basal markers such as CK5/6 and EGFR. Moreover, the solid pattern of classic ACC and some special subtypes of ACC, including solid-basaloid ACC and high-grade transformation of ACC, may also lead to confusion with BL-TNBC. Other studies demonstrated similar transcriptomic characteristics between ACC and BL-TNBC (7). Despite substantial overlapping features with BL-TNBC, ACC has an indolent clinical behavior (8). Thus, it is significant to differentiate ACC from BL-TNBC in order to avoid unnecessary treatment such as radical surgery and excessive chemotherapy.

Recent studies have shown the recurrent t (6;9) (q22–23; p23–24) translocation in salivary and breast ACC results in a novel gene fusion of the MYB proto-oncogene with the transcription factor gene NFIB, and this gene rearrangement has become the major oncogenic event in ACC (9, 10). In addition, subsequent research less commonly found MYBL1-NFIB rearrangements or MYB amplification (11, 12). Given the comparatively high MYB protein expression and low incidence of MYB translocation, some studies argued that MYB activation *via* gene rearrangement or other mechanisms is a diagnostically useful biomarker of ACC (13).

Most of the genomic analysis of ACC was concentrated in salivary ACC with only several small cohorts of breast ACC research. Besides, most of the previous studies explored a limited number of suspected DNA sequencing, mRNA expression, mutational burden, and copy-number alteration (14–16), which has inadequate information to differentiate ACC from BL-TNBC. Proteins are the real executioner of functions encoded in the genome; they play a key role in tumorigenesis and progression and in providing new therapeutic targets (17). Due to recent technological development in liquid chromatography–mass spectrometry (LC-MS) equipment, MS-based proteomics has become a powerful method for the quantitative genome-scale signature of the proteome. The quantitative proteomic analysis is a useful complementary analysis to genomic and transcriptomic analyses for it provides additional biological information that would have been impossible to get merely by genomics approaches (18). Proteomic analysis has been demonstrated as a useful tool with the potential to identify cancer biomarkers and find new therapeutic targets in salivary gland ACC, whereas the detailed information on breast ACC is unknown (19–21).

Based on the limited proteomic research on breast ACC and the urgent need for discriminating ACC from BL-TNBC, we present the first comparative proteomic analysis of breast ACC and BL-TNBC by measuring 5 pairs of tumors and adjacent normal breast tissues. As a result, there is a clear separation between ACC and BL-TNBC on different expression proteins (DEPs) with special functional characteristics. Our study provides functional context to differentiate ACC from BL-TNBC and offers a rich resource for data mining and guidance for clinical validation.

## Materials and methods

### Case selection

For this retrospective study, 10 breast ACC were retrieved from the archival files of the pathology department, Peking University Cancer Hospital, between 2011 and 2020. Due to the extremely low incidence, we can only obtain formalin-fixed paraffin-embedded (FFPE) materials, not frozen tissue, and thus, the subsequent studies were carried out on FFPE specimens; 5 age-matched BL-TNBC were also retrieved and served for proteome comparison. All cases were reviewed and confirmed by two pathologists (QY and DFN). Clinicopathological information and follow-up data were retrieved and analyzed. Follow-up data were collected up to February 2022. Our study obtained permission from the Peking University Cancer Hospital Institutional Review Board and Ethics Committee (Grant: 2021KT29).

### Immunohistochemical staining

Commercially available primary antibodies for AR/CK5/6/CK7/CyclinD1/Ki67/P16/P63/RB (Zhongshan Company, Beijing, China), P53/PTEN/S100/SOX10 (GeneTex, CA, USA), CD117/ER/PR/HER2 (Roche, Basel, Switzerland), and EGFR/CK8/18 (XiYa Reagent, Chengdu, China) were applied. MYB antibody (1:200, Clone ab4515, Abcam, Cambridge, UK), ITGB4 (1:250, Clone ab182120, Abcam, Cambridge, UK), VCAN (1:100, Clone ab177480, Abcam, Cambridge, UK) and DPT (1:50, Clone 10537-1-AP, Proteintech Group, IL, USA) were also used. Immunohistochemical stains were performed on Dako ASL48 platform (Dako, Glostrup, Denmark), following the vendor's protocol. PD-L1 expression was detected using the PD-L1 IHC 22C3 pharmDx kit (Agilent, Santa Clara, CA, USA). The appropriate positive and negative controls were performed on each antibody. The immunohistochemistry (IHC) results were analyzed by 2 pathologists (QY and DFN) with the semiquantitative scoring criteria: 0, ≤25% tumor cells stained; 1+, 26%−50% tumor cells stained; 2+, 51%−75% tumor cells stained; 3+, ≥76% tumor cells stained. For ITGB4, VCAN, and DPT, the staining intensity was graded as 1 (weak or no expression), 2 (moderate expression), and 3 (strong expression), and the intensity and proportion scores were multiplied together to produce a weighted score for each case. Mutation-type labeling of PTEN and RB expressions represented by no expression of cells, otherwise, was wild type. For Ki67, the precise cell proportion was recorded. PD-L1 expression was evaluated by IHC combined positive score (CPS), and CPS≥1 was viewed as positive.

### Fluorescence *in situ* hybridization

Fluorescence *in situ* hybridization (FISH) was carried out by a commercial MYB dual-color break-apart probe kit (CL-003; Wuhan HealthCare Biotechnology Co., Ltd, Wuhan, China). The probe recognizes translocations of the chromosomal region 6q23.3 including the MYB gene. Two probes labeled with green and orange fluorochromes hybridize at the 5′ and 3′ ends of the MYB gene. FISH was analyzed by a trained pathologist (DFN) according to the previous criteria (22).

### Sample preparation

In total, 10 cases of breast ACC were included in the proteomic analysis; however, only five cases were qualified after SDS-PAGE separation and quantification; 15 slices of serial 5 μm FFPE sections were prepared for each case with more than 1 cm^2^ of tumor cells. Samples were lysed in SDT buffer (4% SDS, 100 mM Tris-HCL, pH 7.6) and then sonicated followed by boiling for 15 min. After being centrifuged at 14,000 g for 40 min, the supernatant was quantified by Bradford protein assay. Extracts of 20 μg of proteins for each sample were mixed with 6× loading buffer, respectively, and boiled for 5 min. The proteins were separated on 12% SDS-PAGE gel; 200 μg of proteins for each sample was reduced with 10 mM dithiothreitol at 56°C for 30 min. The samples were digested using the FASP method with trypsin, and the resulting peptides were collected as a filtrate.

### LC-MS/MS analysis

The peptides were separated on C18 cartridges and then concentrated and dried by vacuum centrifugation. The peptide content was accessed by UV light spectral density at 280 nm. LC-MS/MS analysis was conducted on a Q Exactive Plus mass spectrometer (Thermo Fisher Scientific) and Easy nLC (Thermo Fisher Scientific); 2 μg of the peptide was loaded on the C18 reversed-phase analytical column (Thermo Fisher Scientific). MS data were acquired by up to 20 data-dependent methods with higher energy collision dissociation. The ions with a charge state between 2 and 6 and a minimum intensity of 2e3 were qualified for fragmentation. Dynamic exclusion time was set as 30 s. The normalized collision energy was 27 eV.

### Data analysis

The MS data were processed using the MaxQuant search engine (version 1.6.17.0). Trypsin/P was the cleavage enzyme allowing up to two missing cleavage sites. The first search was set at a precursor mass tolerance of 20 ppm and 4.5 ppm in the main search. Carbamidomethylation of cysteines was specified as a fixed modification. Acetylation on protein N-terminal and oxidation on methionine were defined as variable modifications. The false discovery rate (FDR) for peptide and protein identification was adjusted to <1%. Protein abundance was accessed using the normalized spectral protein intensity (LFQ intensity). Proteins with fold change >2 or <0.5 and *p*-value (Student's *t*-test) <0.05 were defined as DEPs. We applied K-nearest neighbor (KNN) imputation by producing an unbiased calculation of the missing values, and we conducted standard batch correction and normalization in R (version 4.1.2). Principal component analysis (PCA) was performed to visualize the difference between tumors and adjacent tissues (Supplementary Figures 1A–C).

### Bioinformatics analysis

The bioinformatics analyses were performed using R (version 4.1.2). “DEP” package was used to process proteomics data, and “clusterProfiler” package was used to process and visualize the DEPs. Gene Ontology (GO) annotation, including biological processes (BPs), cellular compartments (CCs), and molecular functions (MFs), and Kyoto Encyclopedia of Genes and Genomes (KEGG) pathway were performed for the enrichment analysis of gene expression. Gene Set Enrichment Analysis (GSEA) was also used for gene enrichment analysis, and the pathway enrichment was analyzed from the Molecular Signatures Database (MSigDB). Protein-protein interaction (PPI) network construction was conducted using the STRING database (https://string-db.org) with the DEPs identified by proteomic data as input, and the combined score >0.4 was regarded as statistical significance interaction.

## Results

### The clinicopathological characteristics

**Figure 1:**
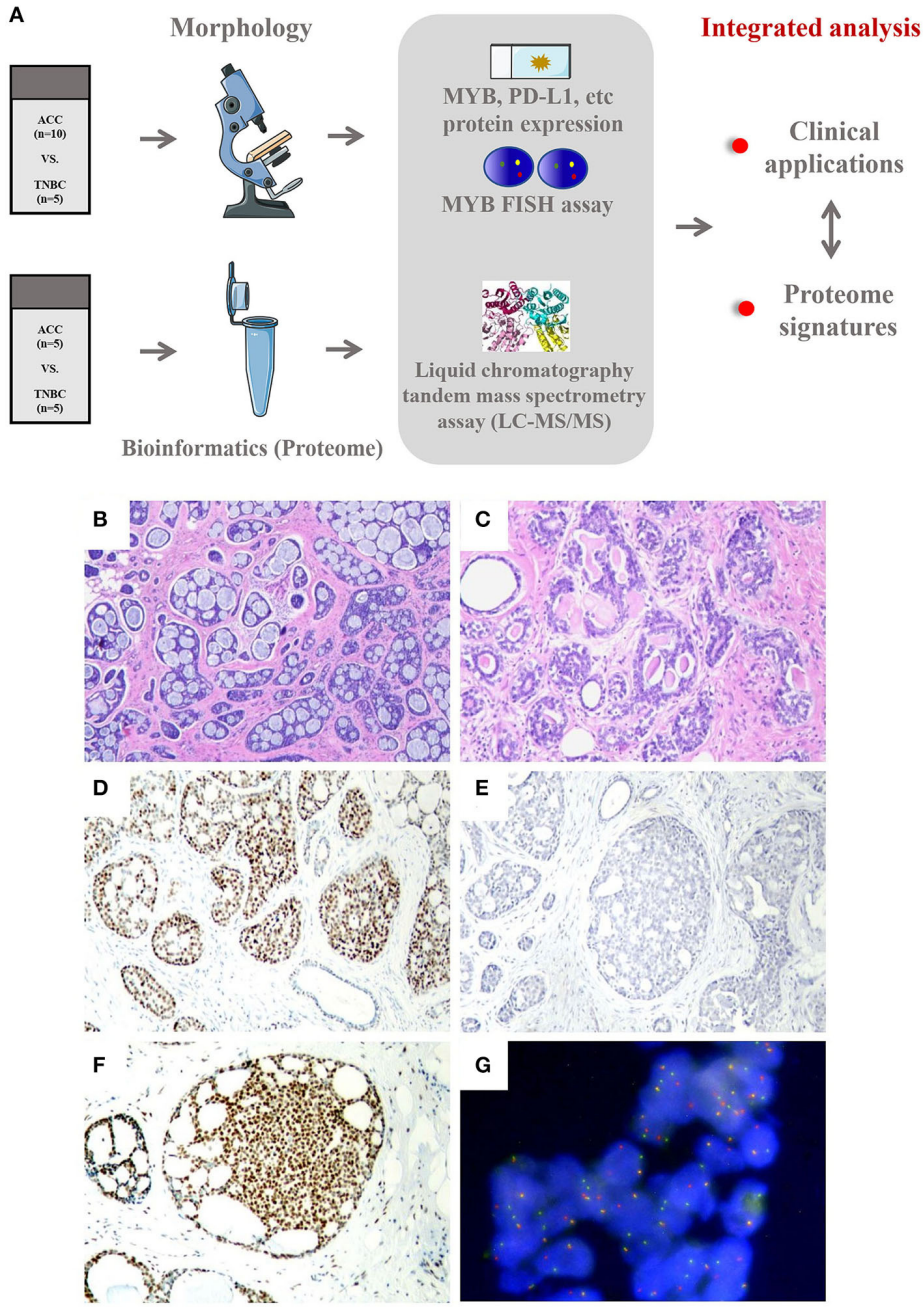
**(A)** Workflow of integrated analysis of ACC compared with BL-TNBC. **(B–G)** Histological, immunohistochemical, and MYB FISH results of ACC. **(B)** Adenoid cystic carcinomas predominantly demonstrating cribriform and tubular architecture (40×). **(C)** The basement membrane material inside the luminal of ACC gland (100×). **(D)** ACC is immunoreactive for Sox10 (100×). **(E)** ACC is completely negative for PD-L1 expression (CPS = 0 on Dako 22C3 platform) (100×). **(F)** ACC showing strong nuclear immunoreactive for MYB (100×). **(G)** Rearrangements of MYB detected by FISH using dual-color break-apart probe (400×).

The workflow of our study is shown in Figure 1A. The clinicopathological parameters of all cases are given in Table 1. Among the 10 cases of ACC, the mean age of patients at diagnosis was 53.7 years (range, 38–78 years). Following surgical resection as the primary treatment, 2 (20%) patients received observation alone and 8 (80%) patients received chemotherapy, while 4 (40%) patients received radiation additionally. The mean follow-up was 5.3 years (range, 1.5–10.5 years). One case of ACC showed local recurrence, which occurred in axillary lymph nodes. All patients were alive without disease. The mean age of BL-TNBC was 55.3 years (range, 32–73 years). All the patients undertook surgery with chemotherapy with or without radiation. The mean follow-up was 1.92 years (To ensure the quality of tissue to perform proteome base analysis, we selected the newly diagnosed cases in our study). Two cases witnessed axillary lymph nodes metastasis. None of the cases showed local recurrence in this short follow-up period. All the patients were alive without disease.

**Table 1:** Clinicopathological characteristics of 10 cases of breast ACC and five cases of BL-TNCB.

**Case**	**Age**	**Size**	**Histological**	**Pathologic**	**Local**	**Treatment**	**Follow-up**	**Outcome**
	**(years)**	**(cm)**	**grade**	**stage**	**recurrence**		**(years)**	
ACC1	40	3.4	1	pT2N1aM0	Yes	Surgery/Chemo/Radiation	9.2	Alive
ACC2	44	2.8	1	pT2N0M0	No	Surgery/Chemo	8.6	Alive
ACC3	53	5.0	1	pT2N0M0	No	Surgery/Chemo	6.1	Alive
ACC4	69	1.1	1	pT1bN0M0	No	Surgery/Chemo/Radiation	2.8	Alive
ACC5	38	4.0	2	pT2N0M0	No	Surgery/Chemo	1.5	Alive
ACC6	43	1.2	1	pT1bN0M0	No	Surgery/Chemo/Radiation	10.5	Alive
ACC7	78	1.1	1	pT1bN0M0	No	Surgery/Chemo	5.5	Alive
ACC8	77	2.0	1	pT1cN0M0	No	Surgery	4.7	Alive
ACC9	41	4.5	1	pT2N0M0	No	Surgery	4.5	Alive
ACC10	54	2.0	2	pT1cN0M0	No	Surgery/Chemo/Radiation	3.0	Alive
TNCB1	32	1.0	3	pT1bN0M0	No	Surgery/Chemo	2.0	Alive
TNCB2	60	2.0	3	pT1cN1aM0	No	Surgery/Chemo/Radiation	2.1	Alive
TNCB3	63	2.0	3	pT1cN3M0	No	Surgery/Chemo/Radiation	1.5	Alive
TNCB4	48	2.5	3	pT2N0M0	No	Surgery/Chemo	2.5	Alive
TNCB5	73	2.3	3	pT2N0M0	No	Surgery/Chemo	1.25	Alive

**Figure 2:**
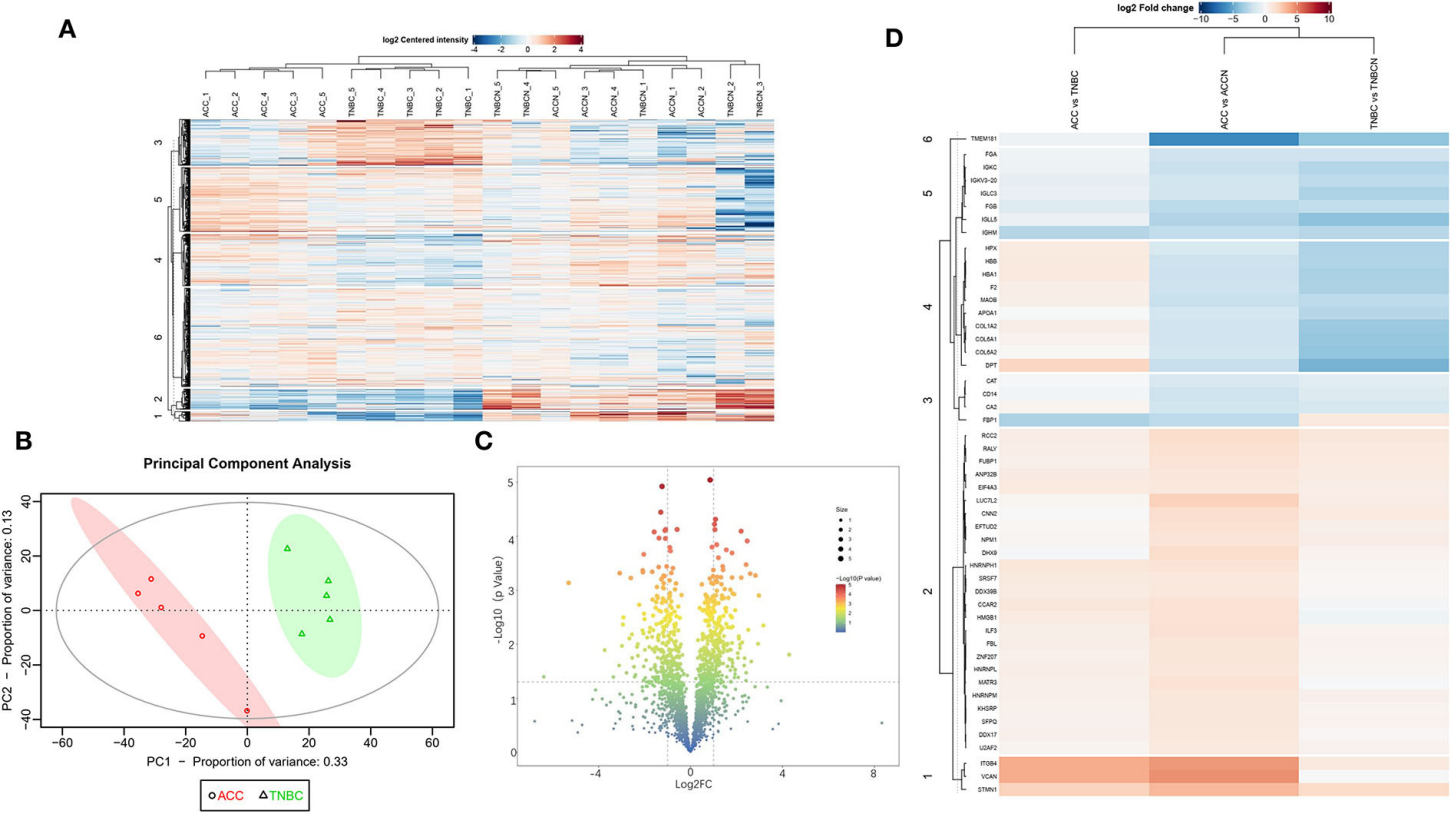
**(A)** Heat map of all DEPs among ACC, BL-TNBC, and the adjacent normal breast tissue. **(B)** PCA of the proteomic data from ACC (red) and BL-TNBC (green). **(C)** Volcano plots of the proteomic analysis showing the variance in expression between ACC and BL-TNBC. The dotted vertical lines indicate one standard deviation from the mean fold change. **(D)** Heat map of top 50 DEPs between ACC and BL-TNBC, ACC, and adjacent normal breast tissue, as well as BL-TNBC and adjacent normal breast tissue. The detailed DEPs are listed on the left line.

Histologically, eight cases showed grade 1 architecture and two cases showed grade 2, according to the Nottingham grading (23). ACC has been traditionally divided into 3 morphological groups, namely, cribriform, tubular, and solid (24). In our cases, 8 (80%) mainly showed cribriform arrangement with a small portion of tubular pattern (Figure 1B) and 2 (20%) showed a solid pattern. The tumor cells are small and round, with less cytoplasm and low mitotic activity. The characteristic collections of basement membrane material were observed in 7 (70%) cases (Figure 1C) and were mostly limited to the cribriform pattern. All BL-TNBC cases showed grade 3 histological grading with a predominant solid growth pattern, as well as outstanding atypia and high mitotic activity. The basement membrane material was absent in BL-TNBC.

### The immunohistochemical characteristics and FISH results

Like BL-TNBC, almost all ACCs have a negative expression for ER, PR, and HER2 and a positive immunoreactivity for CK 5/6 and EGFR; 2 ACC with ER positivity exhibited only weak staining with 5–10% proportion. Among tumor cells, the luminal cell was positive for CK7 (3/3,100%) and CK 8/18 (5/5, 100%), and the myoepithelial-basal cell showed positivity for P63 (8/9, 89%), CK5/6 (9/9, 100%), EGFR 8 (5/5, 100%), and S100 (4/6, 67%). P53 expression was performed on all ACC cases; however, none of them demonstrated mutation type. Other IHC results are summarized in Table 2. As a new diagnostic biomarker for breast ACC (25), Sox10 showed diffuse nuclear positivity in most of the ACCs (9/10, 90%) (Figure 1D) with only weak and focal expression in BL-TNBC. Consistent with the findings of previous literature, a few ACCs express AR (1/10, 10%) and P16 (4/10, 40%) in a weak pattern; the Ki67 index was low with an average index of 12.7% (range, 2%−20%). The AR expression (strong positivity in 60% cases) and P16 expression (strong positivity in 100% cases) were relatively high in BL-TNBC with an increased ki67 proliferation rate (average 68%, range, 60%−80%). We performed a PD-L1 immunohistochemistry assay on ACC, and CPS results showed 0 in 60% (6/10) cases (Figure 1E) and <1 in the rest cases. With the cutoff as 1 in most literature on tumor tissue, our results implied that all ACC were negative on PD-L1 expression. BL-TNBC demonstrated a much higher CPS with a mean index of 36 (range, 15–60). MYB immunohistochemical staining was positive in 70% (7/10) of cases with 40% (4/10) in the strong model (Figure 1F) and 30% (3/10) in the weak model. When we performed FISH assay, 50% (5/10) showed MYB rearrangement (Figure 1G) and the other 20% (2/10) cases demonstrated MYB amplification. None of the BL-TNBC showed MYB positive or MYB rearrangement.

**Table 2:** Immunohistochemistry results for breast ACC and BL-TNBC.

**Case**	**MYB**	**MYB rearrangement**	**AR**	**SOX10**	**CD117**	**PTEN** ^a^	**RB** ^a^	**CyclinD1**	**P16**	**PD-L1**	**Ki67**
ACC1	3+	Yes	0	3+	3+	WT	WT	2+	0	CPS = 0	15%
ACC2	3+	Yes	0	2+	3+	WT	WT	2+	0	CPS <1	20%
ACC3	1+	Yes	1+	2+	2+	WT	WT	1+	1+	CPS = 0	2%
ACC4	3+	Yes	0	1+	2+	MT	WT	1+	0	CPS = 0	10%
ACC5	1+	No	0	3+	3+	MT	WT	1+	0	CPS = 0	10%
ACC6	0	No	0	2+	1+	WT	WT	2+	0	CPS = 0	10%
ACC7	3+	Yes	0	2+	2+	WT	WT	0	0	CPS <1	20%
ACC8	0	No	0	3+	3+	MT	WT	0	1+	CPS = 0	5%
ACC9	1+	No	0	3+	1+	WT	MT	1+	1+	CPS <1	15%
ACC10	0	No	0	3+	3+	MT	WT	1+	1+	CPS <1	20%
TNBC1	0	No	3+	0	1+	WT	MT	0	3+	CPS=15	70%
TNBC2	0	No	0	1+	2+	MT	WT	0	3+	CPS = 60	80%
TNBC3	0	No	0	1+	0	WT	WT	1+	3+	CPS = 30	70%
TNBC4	0	No	3+	0	1+	WT	MT	0	2+	CPS = 45	60%
TNBC5	0	No	3+	1+	1+	WT	WT	1+	3+	CPS = 30	60%

*ACC, adenoid cystic carcinoma; TNBC, triple-negative breast cancer; WT, wild type; MT, mutation type*.

*0,≤25% tumor cells stained; 1+, 26%−50% tumor cells stained; 2+, 51%−75% tumor cells stained; 3+, ≥76% tumor cells stained*.

*^**a**^Mutation-type labeling of PTEN and RB expressions represented by no expression of cells, otherwise was wild type*.

### The proteomic profiling of ACC and BL-TNBC

**Figure 3:**
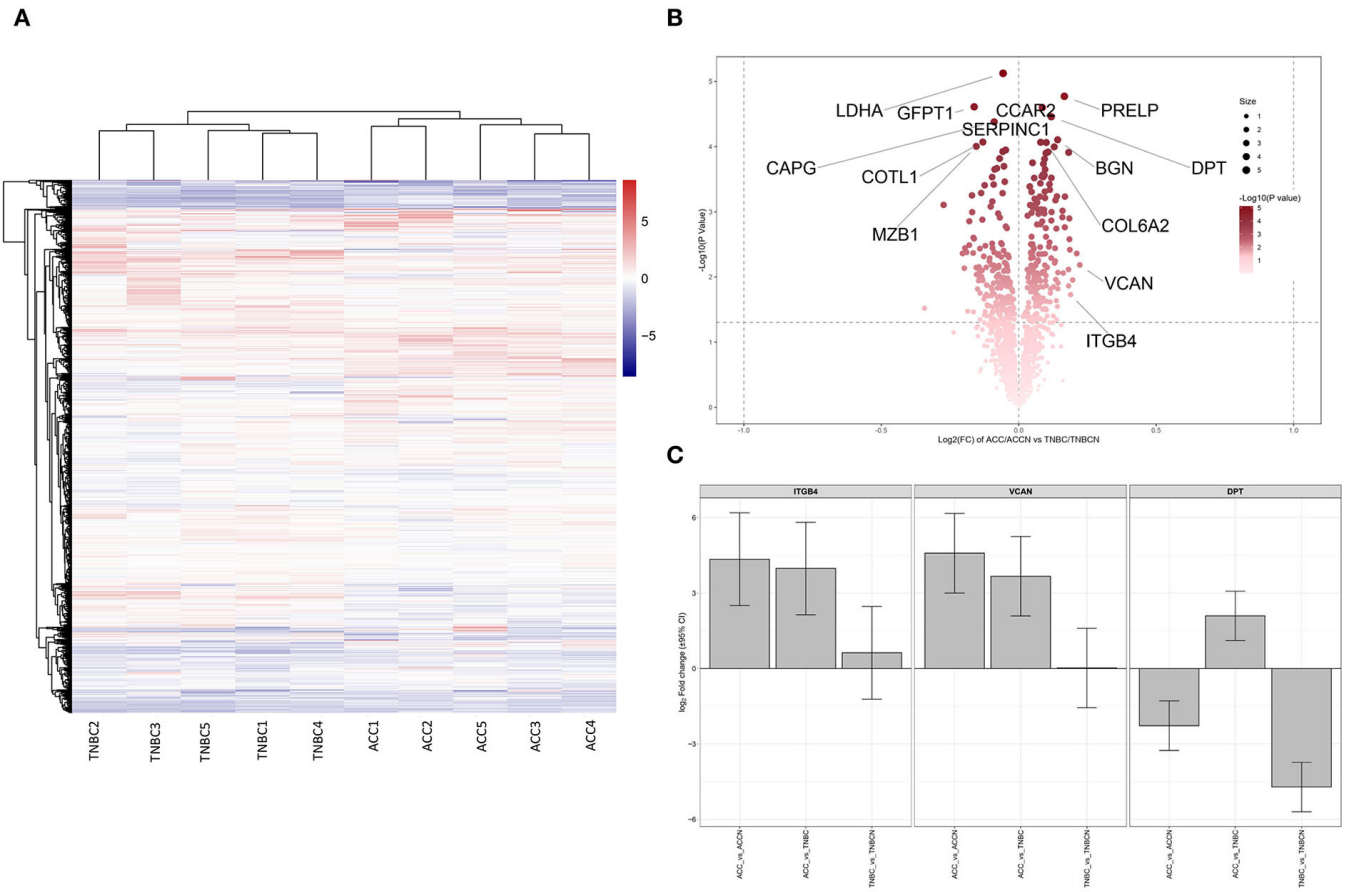
**(A)** Heat map of all DEPs ratio of (ACC/ACCN) and (TNBC/TNBCN). **(B)** Volcano plots of all DEPs ratio of (ACC/ACCN) and (TNBC/TNBCN). Some of the significant DEPs are labeled. **(C)** The expression difference of ITGB4, VCAN, and DPT between ACC and TNBC after normalization by the expression ratio of (ACC/ACCN) and (TNBC/TNBCN). ACCN, the adjacent normal breast tissue of ACC; TNBCN, the adjacent normal breast tissue of TNBC.

**Figure 4:**
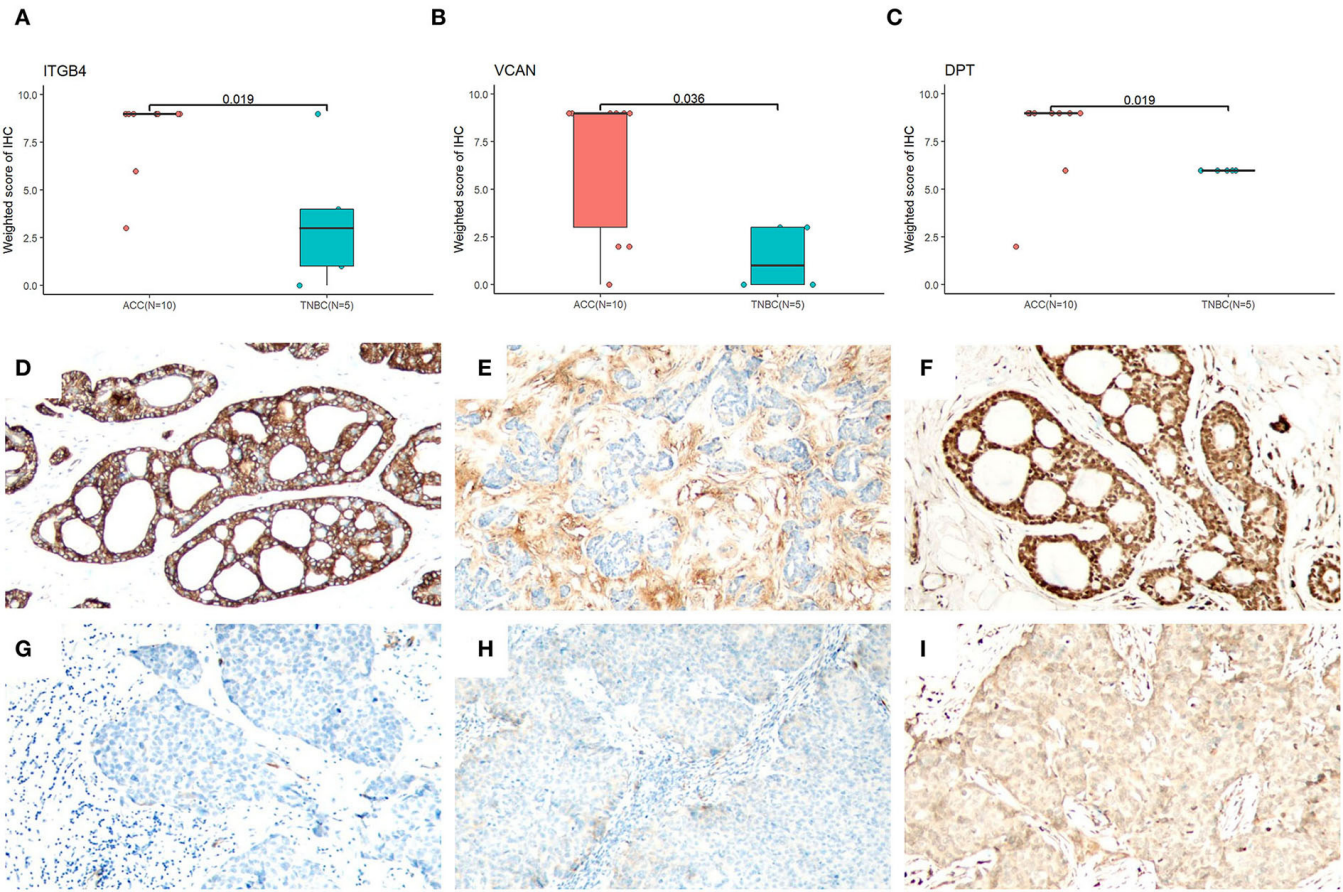
Evaluation of ITGB4, VCAN, and DPT IHC in ACC and BL-TNBC. Statistic results of the weighted score of IHC (Whitney *U* test) for ITGB4 **(A)**, VCAN **(B)**, and DPT **(C)**. **(D)** ITGB4 showing strong membranous expression in ACC (100×). **(E)** VCAN showing diffuse expression in peritumoral stroma of ACC (100×). **(F)** DPT showing strong cytoplasmic and nuclear expression in ACC (100×). **(G)** Completely negative ITGB4 expression in BL-TNBC (100×). **(H)** No VCAN staining in peritumoral stroma staining in BL-TNBC (100×). **(I)** Weak cytoplasmic expression of DPT in BL-TNBC (100×).

Based on the proteomic analysis of ACC and adjacent normal tissue, 2,128 proteins were identified within 19,359 unique peptides, and 196 DEPs were quantified. Similarly, 380 DEPs were quantified from BL-TNBC and adjacent normal tissue, and 307 DEPs were quantified from ACC and BL-TNBC (Supplementary Table 1). All DEPs among ACC, BL-TNBC, and adjacent normal breast tissue were hierarchically clustered by using the Z-score LFQ values. This cluster analysis revealed significant differences between the ACC and BL-TNBC (Figure 2A). Principal component analysis (PCA) also showed a clear separation between ACC and BL-TNBC (Figure 2B). Volcano plots of the proteomic data indicated the difference in expression between ACC and BL-TNBC (Figure 2C). We also compared the top 50 DEPs among ACC, BL-TNBC, and adjacent normal tissue; the hierarchical clustering of ACC and BL-TNBC showed the top three DEPs in ACC compared with BL-TNBC, namely, ITGB4, VCAN, and DPT (Figure 2D). To get a better distinction between ACC and BL-TNBC, we calculated the ratio of (ACC/ACCN) vs. (TNBC/TNBCN) and got the new hierarchical clustering shown in Figure 3A. Figure 3A also demonstrated the clear difference between ACC and BL-TNBC. Besides, the volcano plots of the ratio of (ACC/ACCN) vs. (TNBC/TNBCN) also showed the similar DEPs between ACC and BL-TNBC (Figure 3B) with ITGB4, VCAN, and DPT as the top three increased DEPs in ACC compared with BL-TNBC (Figure 3C). We next evaluate ITGB4, VCAN, and DPT expression in ACC and BL-TNBC tissue using the IHC assay. ITGB4 was detected in the membrane, DPT was detected in the cytoplasm and nuclear membrane, and VCAN was expressed in the peritumoral stroma instead of ACC tumor cells. As expected, there were remarkable differences in the expression levels of ITGB4, VCAN, and DPT between ACC and BL-TNBC (Figures 4D–I). The weighted score of IHC confirmed the statistical difference with *p* = 0.019, 0.036, and 0.019, respectively (Figures 4A–C).

### The general features and altered pathways in the ACC proteome in comparison with BL-TNBC

**Figure 5:**
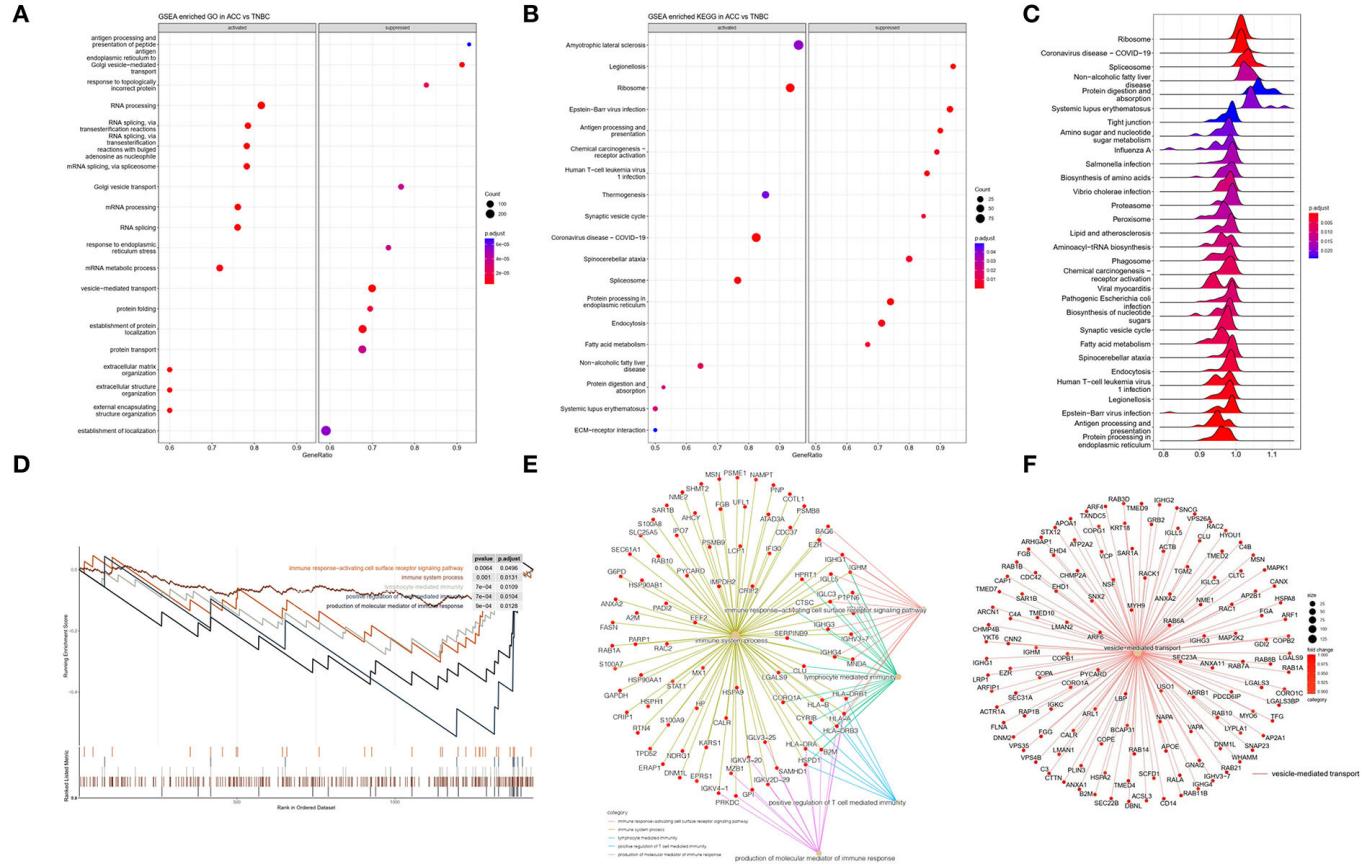
**(A)** GSEA enriched GO analysis in ACC vs. BL-TNBC. GO annotations showing the top activated and suppressed GO terms based on biological process (BP). Significant proteins were included using *p* < 0.05, Benjamini–Hochberg correction (BH), gene set size 10–500, and the whole protein list as the background set. Bubble indicates significant terms by the gradient legend as *p*-adjust < 0.001, and the x-axis refers to gene ratio and the number of proteins contained in given enriched terms. **(B)** GSEA enriched KEGG analysis in ACC vs. BL-TNBC. The top activated and suppressed pathway is shown with the x-axis indicating gene ratio. *p* < 0.05 was considered significant. **(C)** GSEA enriched ridge plot in ACC vs. BL-TNBC. **(D)** GSEA enrichment plots of the suppressed immune response in ACC. **(E)** GSEA enrichment network of the downregulation of immune response in ACC. **(F)** GSEA enrichment network of the downregulation of vesicle-mediated transport in ACC.

We performed GO annotation analysis to functionally classify DEPs observed in the proteomics data. The analysis of the biological process (GO-BP) showed that relative to TNBC, ACC proteins were functionally upregulated in RNA processing, RNA splicing, and extracellular matrix organization; however, they showed downregulation in antigen processing and presentation of peptide antigen, endoplasmic reticulum to Golgi vesicle-mediated transport, Golgi vesicle transport, and vesicle-mediated transport (Figure 5A). In KEGG pathway analysis, we found that compared with BL-TNBC, ACC showed the upregulation of ribosome, spliceosome, and protein digestion and absorption and the downregulation of antigen processing and presentation, chemical carcinogenesis-receptor activation, and human T-cell leukemia virus 1 infection (Figure 5B).

To further investigate ontologies at the level of functional gene sets, 307 quantified DEPs were compared between ACC and BL-TNBC through GSEA. The GSEA revealed that the ribosome, spliceosome, and protein digestion and absorption were significantly upregulated in ACC, whereas tight junction, proteasome, human T-cell leukemia virus 1 infection, antigen processing, and presentation were significantly downregulated in ACC (Figure 5C). Among the different functional gene sets, we noticed that ACC showed the downregulation of the immune system including immune response-activating cell surface receptor signaling pathway, immune system process, lymphocyte-mediated immunity, positive regulation of T-cell-mediated immunity, and the production of molecular mediator of immune response (*p* < 0.001) (Figure 5D) with detailed and interacted DEPs listed as PPI network (Figure 5E). From the PPI network, we noticed proteins HLA-A, HLA-B, and B2M were all inhibited. These proteins belong to MHC class I molecules and they present peptide antigen to CD8^+^ T cell to evoke an antigen-specific immune response. Their inhibition reflected the down regulation of the CD8^+^ T cell immune response. Moreover, the GSEA of DEPs between ACC and normal breast tissue also indicated a low level of immune response-regulating signaling pathway (Supplementary Figures 2A,C). Another interesting finding was that the vesicle-mediated transport pathway was significantly inhibited in ACC (Figure 5F).

### The functional differences between ACC and adjacent normal breast tissue

**Figure 6:**
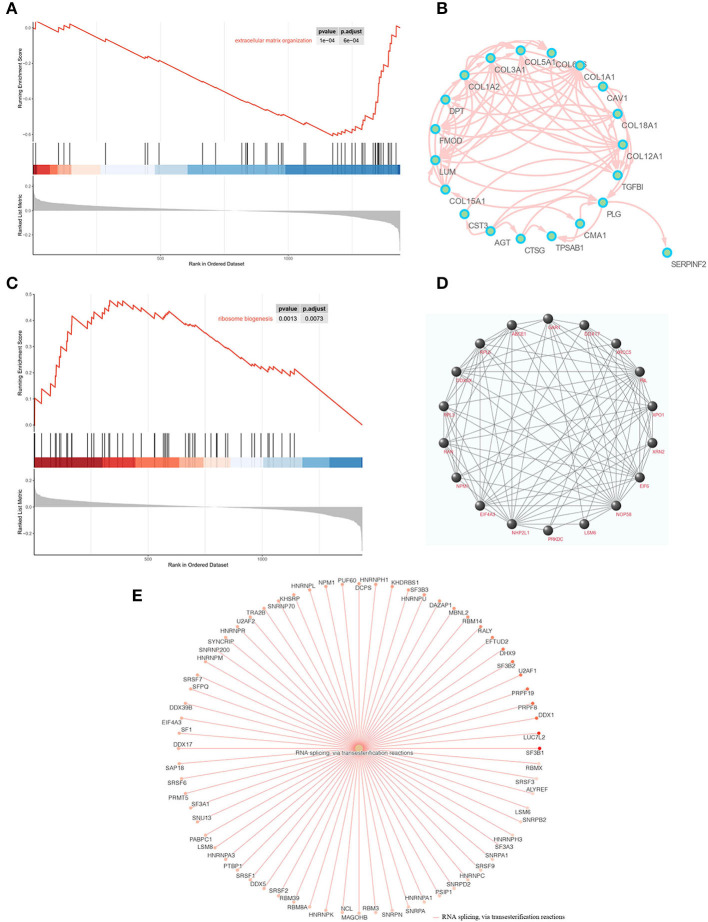
**(A)** GSEA enrichment plots of the downregulation of ECM organization in ACC. **(B)** PPI networks of downregulated biomarkers of ECM organization using STRING v11.5. **(C)** GSEA enrichment plots of the upregulation of ribosome biogenesis in ACC. **(D)** PPI networks of upregulated biomarkers of ribosome biogenesis using STRING v11.5. **(E)** GSEA enrichment networks of the upregulation of RNA splicing in ACC.

We also explored the DEPs between ACC and adjacent normal breast tissue using GO, KEGG, GSEA, and PPI network of DEPs. GO annotations showed the top GO terms based on BPs, CCs, and MFs (Supplementary Figure 2A). The KEGG and GSEA are listed in Supplementary Figures 2B,C. Within the different enrichment pathways between ACC and normal breast tissue, extracellular matrix (ECM) organization was reduced in ACC (Figure 6A); however, it improved in ACC when compared with BL-TNBC (Figures 5A,B). A PPI network was conducted to evaluate the correlation of DEPs in the ECM organization pathway (Figure 6B). Besides, we found that ribosome biogenesis was upregulated in ACC (Figure 5C) with the detailed and interacted DEPs listed as PPI network (Figure 6D). We also demonstrated improved RNA splicing function in ACC (Figure 6E).

## Discussion

The follow-up data of our cases confirmed the indolent nature of breast ACC, consistent with previous studies with 10-year survival over 90% (5, 6). All patients were alive without disease and only one patient had local recurrence in axillary lymph nodes. Our results indicated that the prognosis of breast ACC is much better than BL-TNBC. The morphological characteristics of our cases include the presence of luminal and myoepithelial cells forming 3 architectures, namely, tubular, cribriform, and solid. Only two of the 10 cases have the solid structure, and we failed to correlate the solid pattern with survival owing to the limited cohort. The lumen of the cribriform often contains basement membrane materials. The latter has been demonstrated to represent duplicated basal lamina and glycosaminoglycans by ultrastructural studies (26) and can be used for differential diagnosis including cribriform carcinoma and invasive breast carcinoma not otherwise specified. The American Joint Committee on Cancer Staging Manual (8th edition) recommends that all invasive breast carcinomas should be assigned the Nottingham combined histological grade (23). Thus, our cases belong to grade 1 (8/10, 80%) or grade 2 (2/10, 20%). The result matches with the largest breast ACC study reported to date that breast ACC shares a similar prognosis with grade 1 invasive ductal carcinoma not otherwise specified (5).

According to an immunohistochemical study, our results showed low expression of AR, weak expression of P16, low index of ki67, and wild-type P53 in ACC tissue. These immunohistochemical phenotypes can be used as biomarkers to differentiate ACC from BL-TNBC. Besides, our cases demonstrated negative PD-L1 expression, which was consistent with our proteomic analysis that ACC has a low immune reaction, while PD-L1 expression was highly expressed in BL-TNBC. MYB rearrangement has played a crucial role in the tumorigenesis of breast ACC (9, 10). We found that 70% of cases have nuclear MYB expression but 71.4% of cases have MYB rearrangement using MYB break-apart probe. It means the expression of MYB and the MYB gene mutation were not fully matched, and other factors may influence the MYB protein expression according to previous studies (27, 28). However, we failed to detect MYB overexpression in our proteome analysis nor enrichment of MYB target genes. The undetected proteins with positive FISH arrangement may be due to the low abundance of proteins in FFPE tissue that are below the detection limit of the current method.

Our proteomic data demonstrated the significant difference between ACC and BL-TNBC. GSEA revealed the downregulation of immune response. MHC class I molecules such as HLA-A, HLA-B, and B2M were downregulated in our ACC cases. According to a recent study, MHC class I molecules make an important impact on presenting tumor-specific or tumor-associated peptides to activate CD8^+^ T cell immune response and can be used as the indicator of clinical application of immune-checkpoint inhibitors such as anti-PD-L1 immunotherapy (29, 30). Both proteomic results and PD-L1 expression suggested that breast ACC may have a poor response to immunotherapy, which was in line with the current situation of salivary ACC immunotherapy (31, 32). Our study also highlights the benefits of using proteomic signatures to predict the response to immunotherapy.

The GSEA also revealed that ACC had a significantly downregulated gene set of vesicle-mediated transport by contrast to BL-TNBC. Recently, growing concentration has been laid on the extracellular vesicles, which are defined as nano-sized membrane-bound vesicles released by almost all cells under physiological and pathological processes (33). Referring to TNBC, extracellular vesicles were confirmed to deliver functional materials such as proteins, lipids, mRNAs, non-coding RNAs, and DNA fragments into extracellular location and thus play a significant role in shaping tumor microenvironment and in improving the capability of invasion and metastasis of TNBC (34–36). Our data confirmed higher vesicle-mediated transport activity in TNBC than ACC, which may contribute to elucidate the extreme difference in tumor aggressiveness and prognosis between TNBC and ACC.

Among the top DEPs between ACC and TNBC, ITGB4, VCAN, and DPT were upregulated in ACC, and the expression difference was further confirmed by IHC results. ITGB4 (integrin subunit β4) is a member of the integrins family, which are transmembrane glycoproteins acting as heterodimeric cell adhesion receptors. It has a critical function in connecting the ECM to the cell cytoskeleton (37). VCAN (versican) belongs to the chondroitin sulfate proteoglycan family, which is also one of the major components of ECM and has a vital function in cell adhesion, survival, proliferation, and migration (38, 39). Both ITGB4 and VCAN are considered to correlate with ECM and promote tumor invasion and metastasis of breast cancer with elevated expression (38, 40). Besides, VCAN also correlated strongly with immune suppression and may be responsible for immunotherapy failure (41), which is in line with the suppressed immune response of ACC in our data. DPT (dermatopontin) is a tyrosine-rich ECM protein, which principally takes a significant role in focal adhesion, matrix remodeling, and metastasis of cancer cells (42). The downregulation can accelerate tumor invasion and progression in several types of tumors (43, 44). Our IHC and proteomic data confirmed a high level of DPT expression in ACC while a low level in highly aggressive tumor-BL-TNBC. Therefore, it was hypothesized that DPT might contribute to the favorable biological behavior of ACC. The underlying molecular mechanism of ITGB4, VCAN, and DPT still needs to be further investigated. Similarly, GO, KEGG, and GSEA all suggested that ECM organization pathway was enriched in ACC when compared with BL-TNBC, while it decreased in ACC when compared with normal breast tissue. Given the evidence that components of ECM are highly expressed and promote tumor progression in TNBC and salivary ACC (45–48), our ECM findings suggested that ECM components may play a negative role in the aggressiveness of breast ACC compared with TNBC and salivary ACC and support the distinct biological activity of breast ACC.

When compared with normal adjacent breast tissue, our GSEA reviewed that ACC presents a significantly upregulated pathway in ribosome biogenesis, which is known to be essential for protein biosynthesis and contributes to tumorigenesis (49). Present chemotherapy has already targeted ribosome biogenesis directly to inhibit cell growth and proliferation; however, it leads to severe by-product effects (50). Thus, more precisive target treatments for ribosome biogenesis are still under investigation. The alternative RNA splicing process often correlates with the driver mutation in genes encoding and contributes to cancer progression (51). Breast ACC demonstrated improved activity of RNA splicing in our GSEA results. In line with our data, RNA splicing activity is upregulated in salivary ACC (19, 52). The function of RNA splicing process in breast ACC still needs further study.

## Conclusion

This is the first study to offer integrative proteomic and clinicopathological analysis of ACC compared with BL-TNBC, and our data support the substantially different proteomic profiles between these two neoplasms. ACC exhibits downregulation of immune response and vesicle-mediated transport, while it shows increased activity in ECM organization, ribosome biogenesis, and RNA splicing. The enriched pathway, as well as the detailed overexpressed proteins including ITGB4, VCAN, and DPT, may provide a deep understanding on biological behaviors and potential target therapy of ACC.

## Data availability statement

The datasets presented in this study can be found in online repositories (http://proteomecentral.proteomexchange.org via the iProX partner repository with the dataset identifier PXD033928).

## Ethics statement

The studies involving human participants were reviewed and approved by Peking University Cancer Hospital Institutional Review Board and Ethics Committee. Written informed consent for participation was not required for this study in accordance with the national legislation and the institutional requirements.

## Author contributions

QY: study conception and design, data analysis, and manuscript preparation. WH: research design, statistical analysis, and critical review. JC: proteomic analysis, graphic preparation, and statistical review. ML: data collection and statistical analysis. YB: study conception and critical revision of the manuscript. XH: acquisition of data. CZ: data analysis and manuscript preparation. LZ: acquisition of data and graphic preparation. DN: study conception, manuscript preparation, critical review, and manuscript finalization. All authors contributed to the article and approved the submitted version.

## Funding

This study was supported by the National Natural Science Foundation of China (No. 81301879 and No. 81702839), Incubating Program of Health Committee of Haidian District, Beijing (No. HP2022-31-503001), and Science Foundation of Peking University Cancer Hospital (No. 2021-11).

## Conflict of interest

The authors declare that the research was conducted in the absence of any commercial or financial relationships that could be construed as a potential conflict of interest.

## Publisher's note

All claims expressed in this article are solely those of the authors and do not necessarily represent those of their affiliated organizations, or those of the publisher, the editors and the reviewers. Any product that may be evaluated in this article, or claim that may be made by its manufacturer, is not guaranteed or endorsed by the publisher.

## Supplementary material

Supplementary Figure 1 Quality control of protein data. **(A)** A heat map of proteins with missing values. **(B)** Normalization for all samples before and after normalization. **(C)** The PCA plot for a high-level overview of the data.

Supplementary Figure 2 **(A)** GSEA enriched GO analysis in ACC vs normal breast tissue. GO annotations indicating the top activated and suppressed GO terms based on biological process (BP). **(B)** GSEA enriched KEGG analysis in ACC vs normal breast tissue. The top activated and suppressed pathway is shown with the x-axis indicating gene ratio. p < 0.05 was considered significant. (C) PPI networks of upregulated biomarkers of adaptive immune response and RNA splicing using STRING v11.5.

Supplementary Table 1 All the quantified proteins and DEPs of ACC, BL-TNBC, and adjacent normal breast tissue.
