# Supplementary figures and images for: Comparative proteomic and clinicopathological analysis of breast adenoid cystic carcinoma and basal-like triple-negative breast cancer
Source: Front Med (Lausanne). 2022 Jul 28;9:943887. doi: 10.3389/fmed.2022.943887 (PMC9366086; doi:10.3389/fmed.2022.943887)

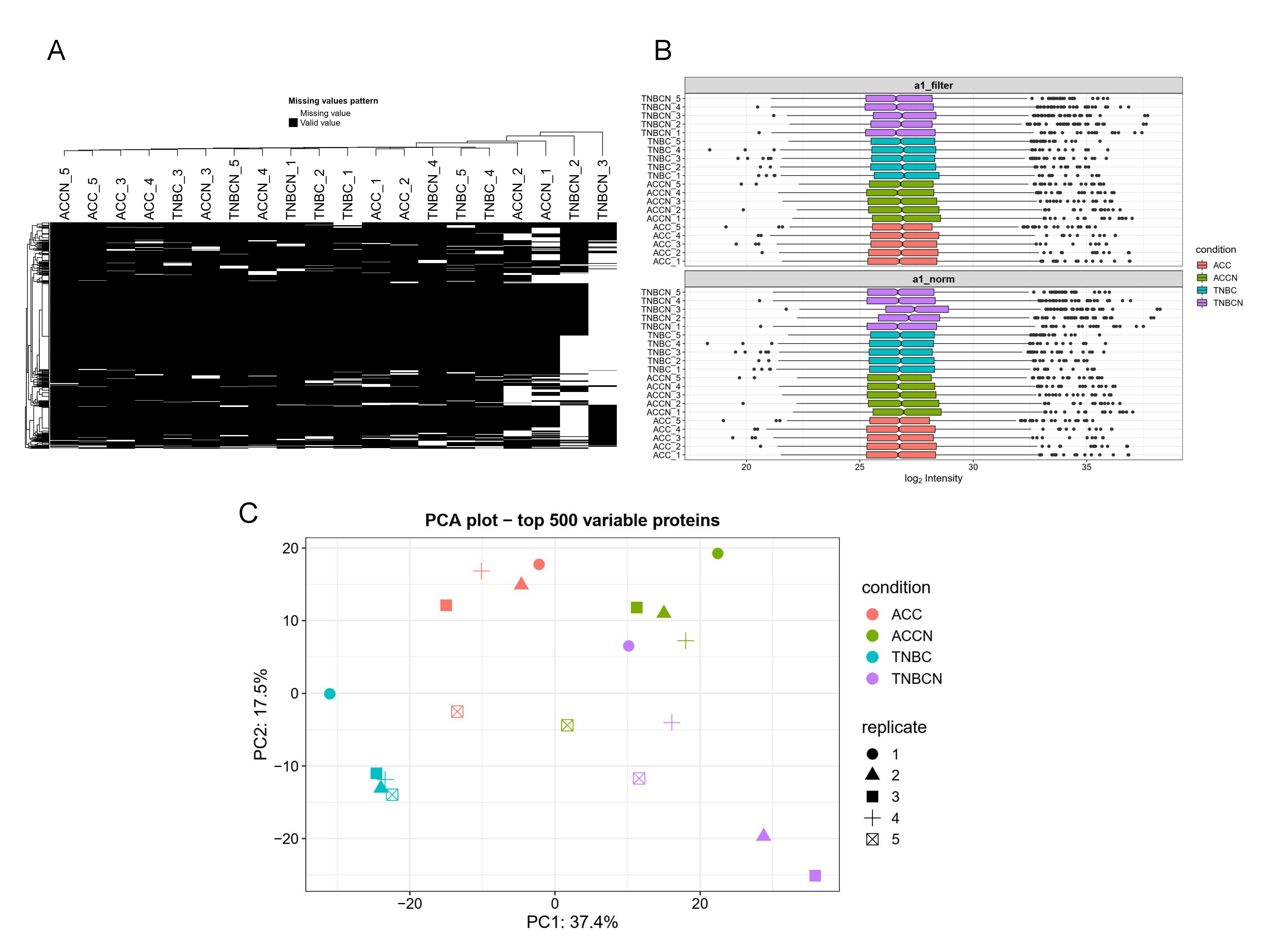

Supplement: Supplementary Figure 1 — Quality control of protein data. (A) A heat map of proteins with missing values. (B) Normalization for all samples before and after normalization. (C) The PCA plot for a high-level overview of the data. [file Image_1.TIF]

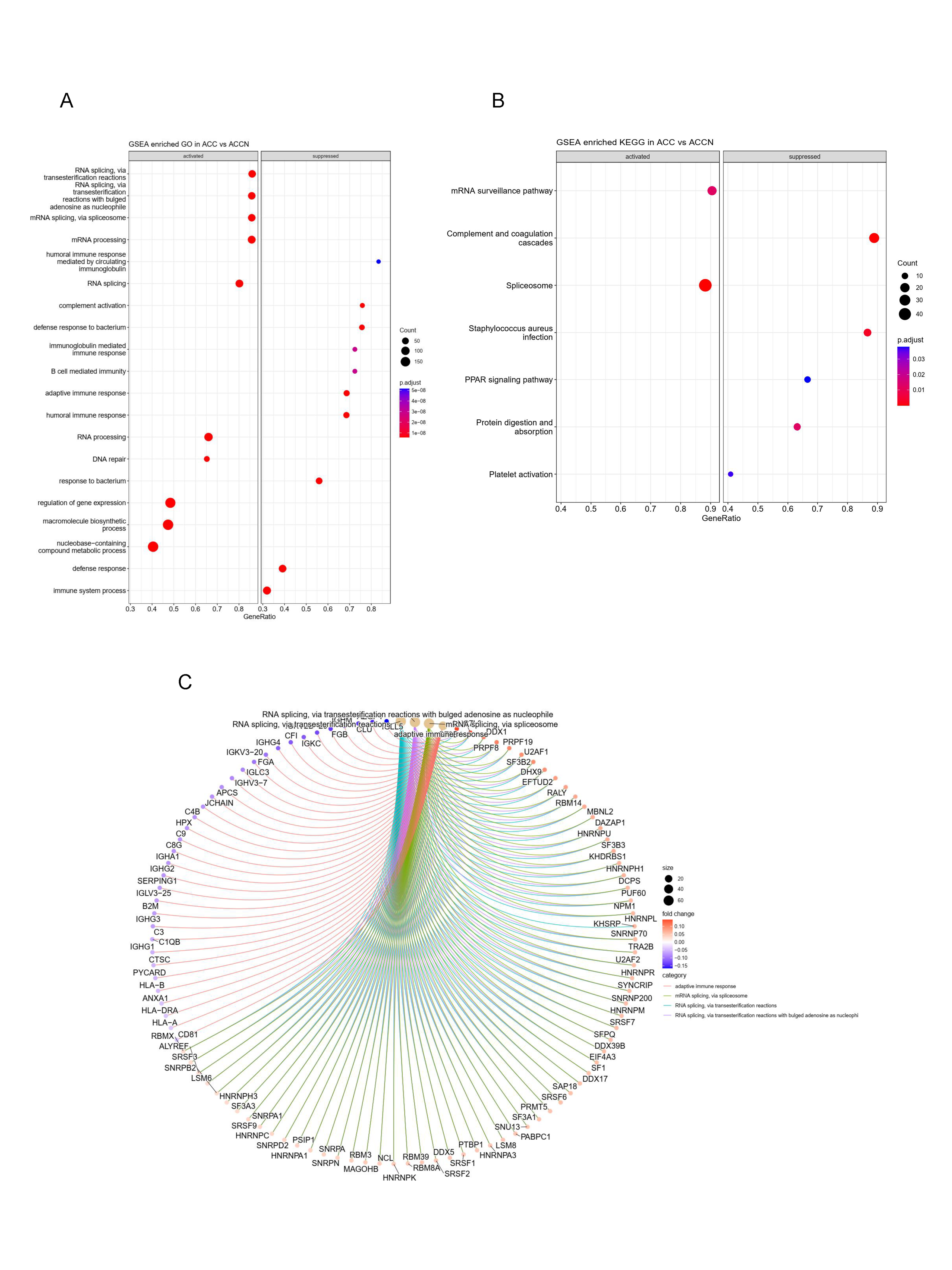

Supplement: Supplementary Figure 2 — (A) GSEA enriched GO analysis in ACC vs normal breast tissue. GO annotations indicating the top activated and suppressed GO terms based on biological process (BP). (B) GSEA enriched KEGG analysis in ACC vs normal breast tissue. The top activated and suppressed pathway is shown with the x-axis indicating gene ratio. p < 0.05 was considered significant. (C) PPI networks of upregulated biomarkers of adaptive immune response and RNA splicing using STRING v11.5. [file Image_2.TIF]
